# Incidental hepatic tuberculosis during planned resection of locally advanced ampullary carcinoma: a case report

**DOI:** 10.1186/s12893-020-00806-8

**Published:** 2020-06-30

**Authors:** Vee Chuan Hoe, Allim Khairuddin, Jun Sam Tan, Mohd Sharifudin Sharif, Nornazirah Azizan, Firdaus Hayati

**Affiliations:** 1 Department of Surgery, Queen Elizabeth Hospital, Ministry of Health Malaysia, Kota Kinabalu, Sabah Malaysia; 2 Gleneagles, Kota Kinabalu, Sabah Malaysia; 3 Department of Pathobiology and Medical Diagnostic, Faculty of Medicine and Health Sciences, Universiti Malaysia Sabah, Kota Kinabalu, Sabah Malaysia; 4 Department of Surgery, Faculty of Medicine and Health Sciences, Universiti Malaysia Sabah, Kota Kinabalu, Sabah Malaysia

**Keywords:** Ampullary carcinoma, Hepatic metastasis, Hepatic tuberculosis, Case report

## Abstract

**Background:**

Tuberculosis (TB) is classified according to the site of disease as pulmonary or extrapulmonary. Extrapulmonary TB is less common than its counterpart in which it can be found anywhere in the body including the liver. Similar to ampullary carcinoma, TB liver can manifest with jaundice and deranged liver function tests, particularly in the obstructed biliary systems.

**Case presentation:**

A 43-year-old gentleman with locally advanced ampullary carcinoma was noticed to have multiple suspicious liver nodules intraoperatively during curative ampulla resection. The surgery was then abandoned after a biopsy. The histology was consistent with chronic granulomatous inflammation. He was then subjected to a Whipple pancreaticoduodenectomy procedure after initiation of anti-tubercular treatment. He recovered well with no evidence of tumour recurrence and worsening TB.

**Conclusions:**

A high index of suspicion and quick decision making can help to diagnose a possible extrapulmonary TB masquerading as a malignant disease in a patient with curative intention of ampullary carcinoma.

## Background

Ampullary carcinomas are neoplasms that arise within the ampulla of Vater, distal to the bifurcation of the common bile duct (CBD) and pancreatic duct. The most prevalent initial presenting symptom is obstructive jaundice, caused by compression of the distal bile duct. Elevated liver function test (LFT) may reflect the site of disease; involvement of the biliary ducts of porta can be reflected by elevated levels of alkaline phosphatase (ALP) and gamma-glutamyl transferase, whereas high transaminases levels may reflect the rare involvement of the liver parenchyma. Furthermore, helical computed tomography (CT) scanning demonstrates local tumour invasion and, more importantly, any presence of distant metastases, which most frequently involve the lymph nodes, peritoneum, and liver [1].

Similar to ampullary carcinoma, tuberculosis (TB) liver can present with jaundice and deranged liver function tests particularly in the obstructed biliary systems [2]. CT imaging in TB liver may demonstrate similar intrahepatic lesions such as macroscopic and microscopic nodules, abdominal lymphadenopathy as well as ascites [3]. Although careful evaluation of associated symptoms and signs can often lead to the correct diagnosis, it is difficult to determine the absolute nature of any liver lesions. We highlight a 43-year-old gentleman with locally advanced ampullary carcinoma for a definitive surgery with an accidental intraoperative finding of TB liver and discuss our approaches.

## Case presentation

A 43-year-old gentleman presented with a 4-month history of painless jaundice with tea-coloured urine and loss of weight. He did not have any fever, night sweats, cough or other respiratory symptoms. He denied taking alcohol, having history of blood transfusion, past medical or family history of malignancy and pulmonary TB contact. On examination, he was icteric with no stigmata of chronic liver disease. There was no abdominal mass or tenderness felt. Biochemical investigation results revealed a serum total bilirubin of 45, direct bilirubin of 31 (normal: 3–22 μmol/L), ALP of 258 (normal: 30–120 mg/dL), and cancer antigen (CA) 19–9 of 2061 (normal: 0–37 U/mL). Besides, inflammatory markers also were obtained showing serum C-reactive protein of 132.5 (normal range: 0–5 mg/L) and erythrocyte sedimentation rate of 106 (normal range: 1–13 mm/Hr). As ultrasound abdomen showed a periampullary mass, an oesophagogastroduodenoscopy was done showing an ulcerated mass at the ampulla. The biopsy was consistent with adenocarcinoma. A staging CT demonstrated a periampullary mass with cystic lesions located at the periphery of the liver suggestive of liver cysts.

**Fig. 1:**
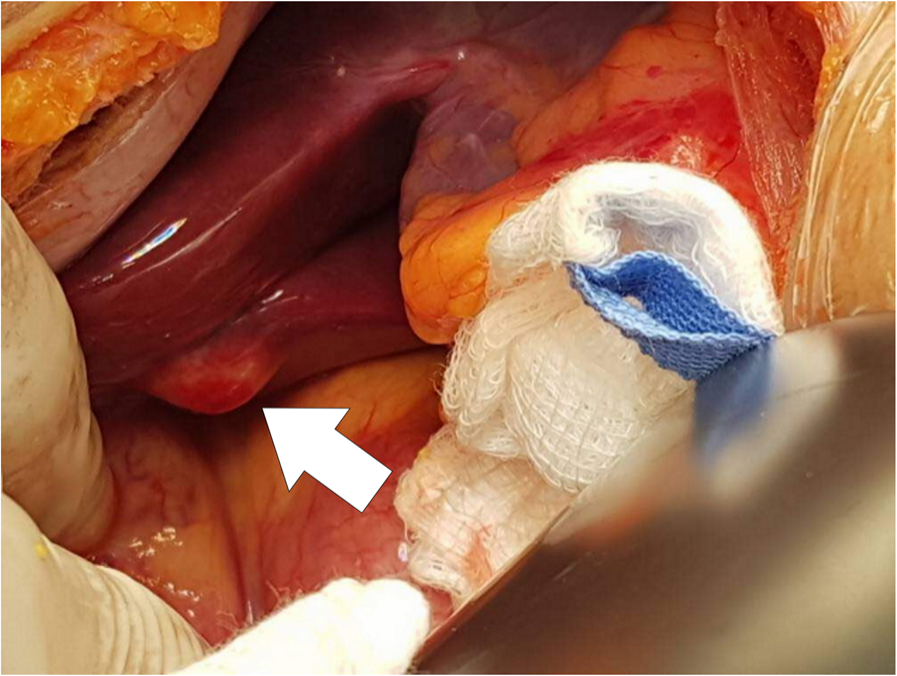
One among the multiple solid nodules (arrow) was identified on the liver surface which was biopsied

**Fig. 2:**
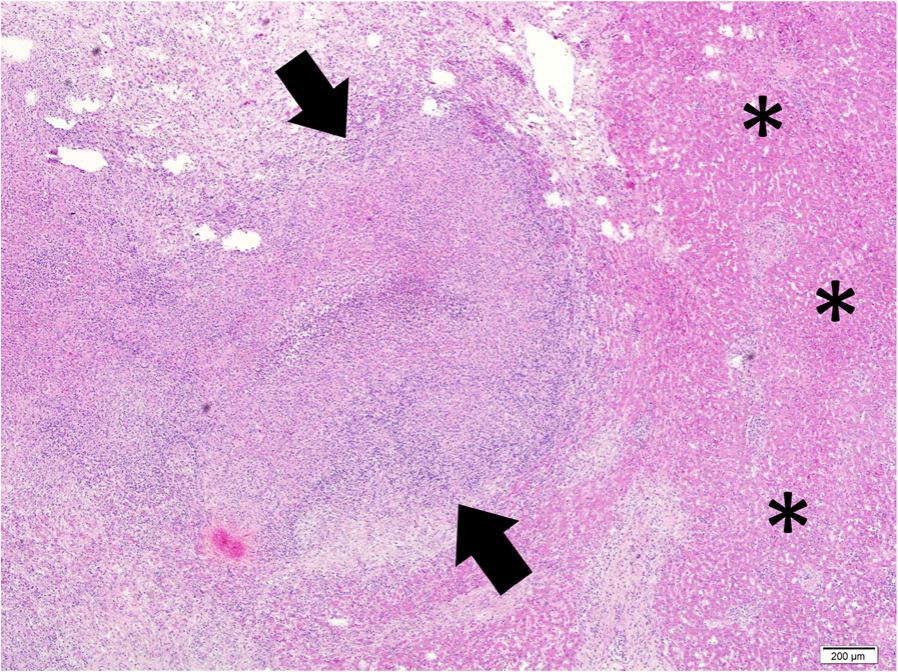
Liver biopsy showing microscopic evidence of granuloma formation (arrow). Note the presence of liver parenchyma at the periphery (*) (Haematoxylin and eosin stain, original magnification × 4)

The patient underwent an elective Whipple pancreaticoduodenectomy procedure with definitive intention to resect the tumour. However, upon entry into the abdominal cavity, there were multiple solid nodules visualized on the surface of both liver lobes (Fig. 1). Since there was no frozen section facility available during that time, an immediate decision was made to abandon the surgery with a possibility of liver metastasis. Hence, a biopsy was undertaken. The biopsy of the liver nodules revealed a chronic granulomatous inflammation giving an impression of extrapulmonary TB (Fig. 2) He was then started on anti-tubercular treatment (ATT) after discussion with the infectious disease team.

**Fig. 3:**
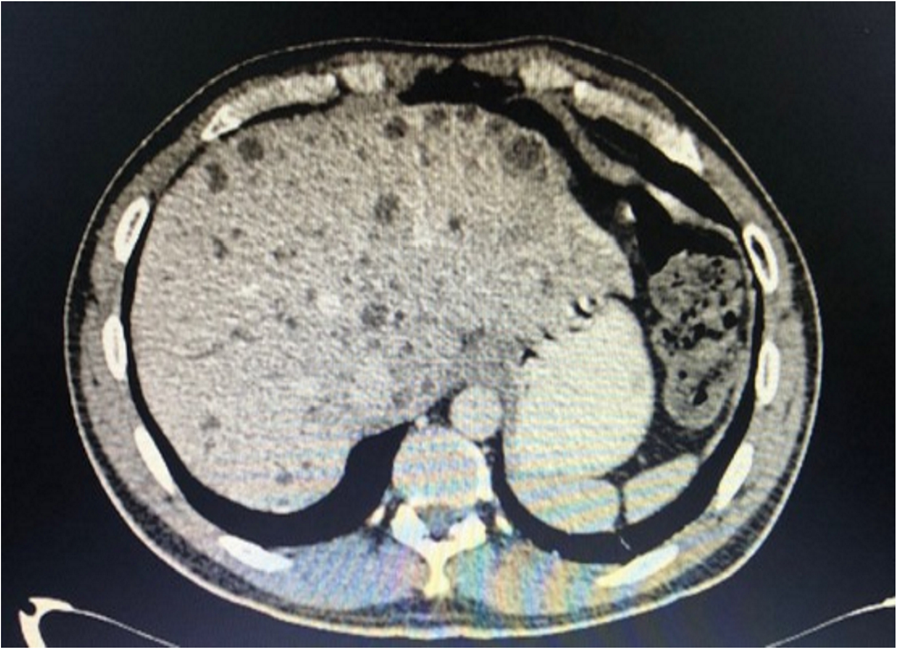
Multiple hypodense hepatic lesions which represent TB liver noted on both sides of liver lobe

**Fig. 4:**
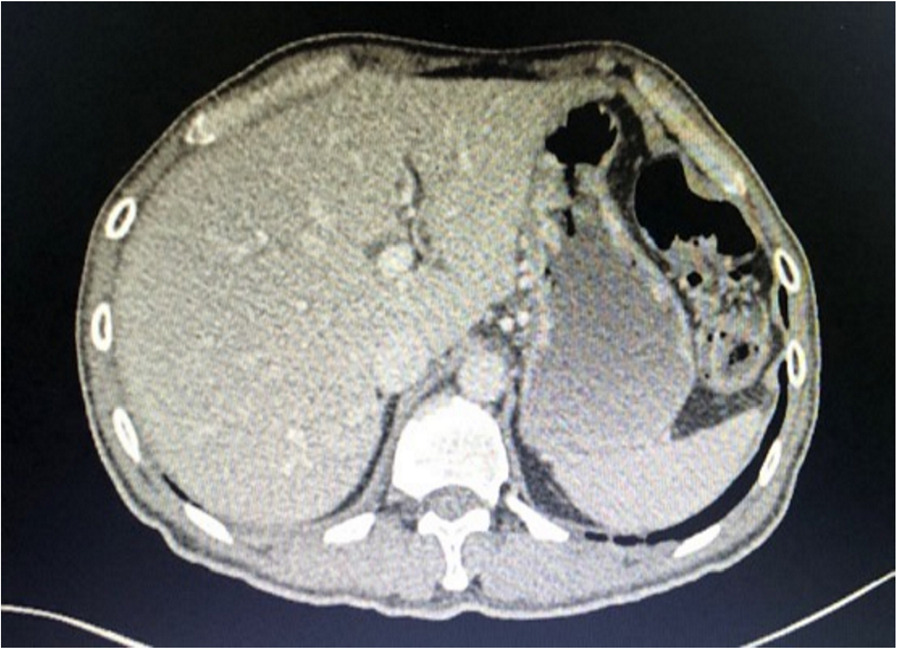
Resolution of TB liver on CT imaging after 6 months post ATT

The patient underwent a Whipple procedure on day 8 of ATT. The surgery went well without any perioperative complications. He was discharged well on postoperative day 10. The histopathological report showed a moderately-differentiated adenocarcinoma with negative resection margins. During follow-up at 1-month post-surgery, repeat CT scan showed no residual lesion within the residual body and tail of the pancreas and the previously seen hypodense hepatic lesions were reduced in number and size (Fig. 3). Subsequent follow-up CT scans demonstrated no local recurrence and absent hepatic lesions (Fig. 4). To support his remarkable recovery, his CA 19–9 had reduced to 65.8 (normal: 0–37 U/mL).

## Discussion and conclusions

The prognosis of ampullary carcinoma is guarded if neglected. The only potentially curative treatment for ampullary carcinoma is Whipple pancreaticoduodenectomy. Although the surgery traditionally has been associated with relatively high negative outcomes, advances in operative techniques, anaesthetic management and vigorous postoperative care have led to significant improvements in outcomes with a 5-year survival rate of 70% [4, 5]. Individuals who have advanced locoregional or metastatic disease, however, carry a poorer prognosis. In fact, the 1-year survival rate after resection of the ampullary tumour and hepatic metastases is found to be 13% with a median overall survival of 5.9 months [6]. Therefore, patients’ selection and cancer staging determination prior to definitive surgery are crucial. In our case, a quick decision was made by getting a tissue biopsy intraoperatively before proceeding to such surgery. If the biopsy turns out to be malignant, the stage of disease will be advanced. Luckily, our biopsy revealed a TB liver.

Tuberculosis is endemic in Asia especially in Malaysia with 11% of the cases of extrapulmonary TB [7]. TB liver is reported to exist in 50–80% of patients who are dying of pulmonary TB [8]. On the contrary, primary TB liver is considered to be rare and constitutes less than 1% of all cases [9]. The spread of hepatic tuberculosis can be attributed by hematogenous spread from active pulmonary TB, swallowing of infected sputum in the active lung disease and ingestion of infected food products. Classification of liver TB has remained in dispute even until now but many authors agree with the existence of the following 3 types namely nodular tuberculosis, military tuberculosis, and mixed tuberculosis [3]. Clinical presentation is extremely varied and often involves abdominal pain, abdominal mass, nausea and vomiting, jaundice, hepatomegaly, ascites, anorexia, and weight loss. This presents a particular diagnostic challenge to clinicians as the diverse features of the disease can mimic many conditions, including malignancy, systemic infections, and viral hepatitis including TB.

TB liver is a treatable disease and the approach to ATT for it is similar to the extrapulmonary TB. A combination of drugs may be given as part of a 9 to a 12-month regimen consisting of 4 first-line drugs (rifampicin, isoniazid, pyrazinamide, and ethambutol) [10]. They are given in 2 phases namely intensive phase (first 2 months) and maintenance phase (next 3 month and beyond) [7]. In order to avoid non-compliance towards ATT, the initiation of fixed dosage such as Akurit-4 and directly observed therapy short-course has improved its delivery.

The current patient case is an explicit example of a rare form of TB masquerading as a liver metastasis occurring with a locoregional ampullary tumour. The correct diagnosis was so important in this case because both aetiologies if treated erroneously, might lead to accelerated morbidity and mortality. In the absence of histopathological examination, the patient may have been misdiagnosed with distant metastasis involvement of the liver and treated differently in view of the poor prognosis that metastatic ampullary carcinoma carries. On the contrary, with the correct diagnosis of both conditions, the patient can be treated with favourable results. Therefore, some investigators have supported the use of diagnostic laparoscopy as a method to stage and assess resectability of metastatic disease prior to resection [11]. This is important because this can minimize hospitalization and postoperative complications.

In conclusion, liver tuberculosis, although rare, can mimic primary or metastatic hepatic tumour. Therefore, a high clinical suspicion is required to recognize this possibility while evaluating ampullary cancer patients with hepatic lesions, especially in TB endemic regions. Thus, early diagnosis and immediate treatment of TB can be initiated and at the same time, any surgical procedure can be carefully planned with fewer complications. However, there are no protocols for this type of case, therefore treatment should be tailored to each scenario.

## Data Availability

Not applicable.

## Abbreviations

TB: Tuberculosis
CBD: Common bile duct
LFT: Liver function test
ALP: Alkaline phosphatase
CT: Computed tomography
CA: Cancer antigen
ATT: Anti-tubercular treatment
